# Association between the initiation of loop diuretics and changes in 2-hour creatinine clearance in critically ill patients

**DOI:** 10.1080/0886022X.2025.2457514

**Published:** 2025-02-04

**Authors:** Kwok M. Ho, Prakkash Ananthan, David J. R. Morgan

**Affiliations:** a Department of Anaesthesia & Intensive Care, Prince of Wales Hospital, The Chinese University of Hong Kong, Hong Kong SAR, China; b Department of Intensive Care Medicine, Westmead Hospital, Sydney, Australia; c Department of Intensive Care Medicine, Fiona Stanley Hospital, Perth, Australia

Current evidence does not support the use of loop diuretics to prevent or treat acute kidney injury (AKI) [1]. Nonetheless, loop diuretics are frequently used to induce diuresis and achieve negative balance in critically ill patients. They are also sometimes used as furosemide-stress test to assess the severity of AKI. However, whether the use of furosemide to induce negative fluid balance in critically ill patients, with and without AKI, is harmful remains uncertain [1, 2].

Theoretically, by inhibiting the Na-K-Cl_2_ cotransporters in the loop of Henle, furosemide may trigger the tubuloglomerular feedback [3, 4], leading to glomerular afferent arteriolar constriction and a reduction in glomerular filtration rate [5]. In patients with heart failure, there is also evidence suggesting that loop diuretics may worsen creatinine clearance [6] and reduce the excretion of a harmful gut microbiota metabolite, Trimethylamine N-Oxide [7].

The association between the initiation of furosemide to induce diuresis and changes in glomerular filtration rate in critically ill patients has not been thoroughly studied. In this prospective cohort study, we aimed to assess whether the initiation of loop diuretics was associated with significant changes in 2-h creatinine clearance (CrCl) within 48 h.

After obtaining approval from the Clinical Safety and Quality Unit of the East Metropolitan Health Services (no: 3142) with waiver of consent, 31 critically ill adult patients who required the initiation of loop diuretics for fluid overload or oliguria due to suspected AKI were recruited (Table 1). Baseline renal function assessment, including a 2-h CrCl and urinary sodium concentration, were obtained before the initiation of diuretics. The dose of diuretics and the use of additional diuretics, if any, were at the discretion of the attending physicians. The same renal function assessments were repeated within 48 h. Patients with end-stage renal failure, AKI on renal replacement therapy, or contraindications to loop diuretics were excluded. There was no missing data on the variables assessed in this study.

**Table 1.** Characteristics of the cohort (*N* = 31).

Variable	Median (IQR) unless stated otherwise
Age, years	56 (44–67)
Male, no (%)	20 (65)
Sepsis, no. (%)	18 (58)
Co-existing medical illness, no. (%)	
Ischemic heart disease	2 (7)
Obstructive airway disease	7 (23)
Peripheral vascular disease	1 (3)
Cirrhosis	1 (3)
Diabetes mellitus	5 (16)
Immunosuppressed	6 (19)
Chronic renal impairment	2 (7)*
Indications to initiate diuretics according to the attending clinicians, no. (%):	
Negative fluid balance	28 (90)
Treatment of acute kidney injury	3 (10)
SOFA score on day of initiating diuretics	6 (4–9)
Use of vasopressor at the time of initiating diuretics, no. (%)	18 (58)
Loop diuretic daily dose (mg)^$^	40 (20–60)
Concomitant use of potassium sparing diuretics, no. (%)	11 (36)
Baseline urea concentration, mmol/L	8.7 (6.5–12.8)
Baseline creatinine concentration, umol/L	68 (50–103)
Baseline urinary sodium concentration, mmol/L	24 (10–53)
Baseline urinary output over 2 h prior to initiation of diuretics, mL	96 (65–150)
Baseline 2-hour creatinine clearance, ml/min	94 (47–133)
Fluid balance on day of initiating diuretics, ml	843 (-84 to +1486)
Day-1 fluid balance, ml	−552 (-1310 to +310)
Day-2 fluid balance, ml	−286 (-858 to +384)
Day-2 urea concentration, mmol/L	9.5 (6.8–15.5)
Day-2 creatinine concentration, umol/L	63 (46–91)
Day-2 urinary sodium concentration, mmol/L	85 (50–121)
Day-2 urinary output over 2 h, mL	340 (167–450)
Day-2 2-hour creatinine clearance, ml/min	42 (18–77)
Number of patients with a reduced 2-hour creatinine clearance after initiation of diuretics (%)	24 (77)
Peak plasma creatinine concentration within 7 days, umol/L	77 (60–141)
Length of ICU stay, days	11 (8–17)

IQR, interquartile range. SOFA, Sequential Organ Failure Assessment. ICU, intensive care unit. *Background plasma creatinine concentrations prior to the onset of critical illness of these two patients were 140 and 165 μmol/L. ^$^Furosemide was used in 30 patients (97%) and ethacrynic acid (50 mg twice per day) was used in one patient who had a history of allergic reaction to furosemide.

**Figure 1.**
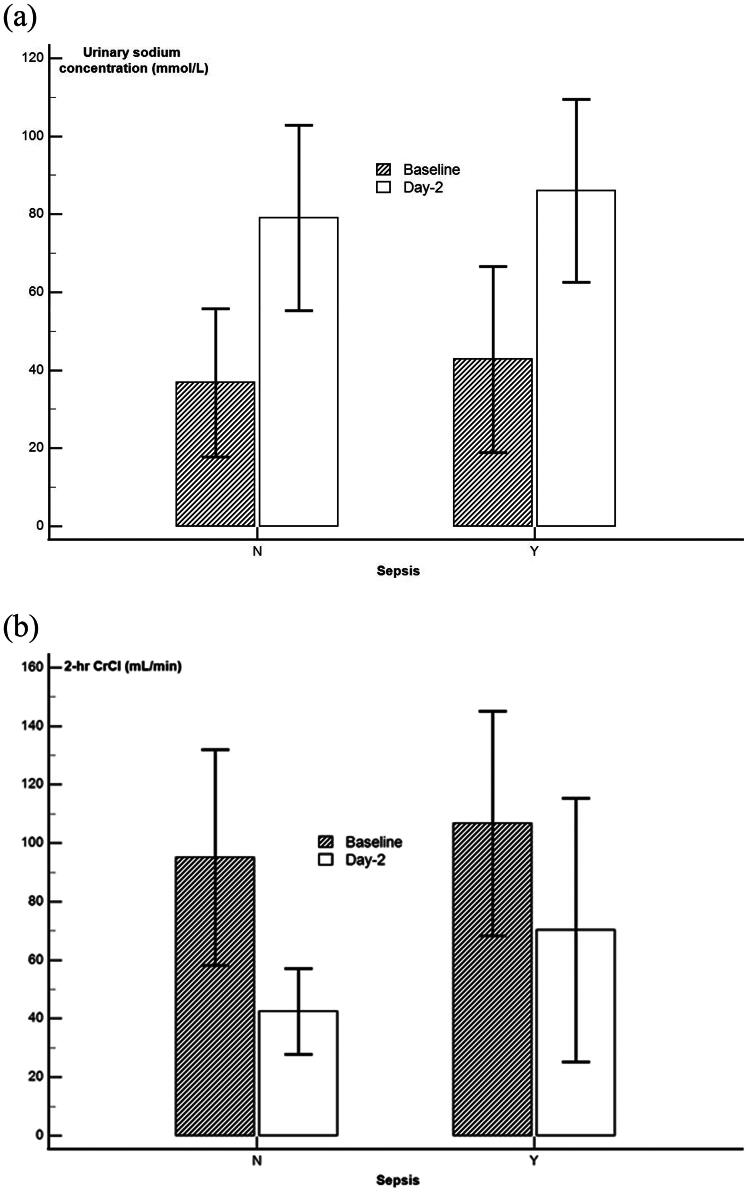
(A) Changes in urinary sodium concentration and (B**)** 2-h creatinine clearance (CrCl) between baseline and day-2 after the initiation of loop diuretics, stratified by whether the patients had sepsis. Data are presented as means with error bars indicating 95% confidence interval.

Among the 31 patients considered, 28 (90%) received loop diuretics (median daily dose: 40 mg, interquartile range [IQR]: 20–60 mg) to induce diuresis and achieve negative fluid balance. Significant negative fluid balance was observed on day-1 (median: −552 mL, IQR −1310 to +310 mL) and day-2 (median: −286 mL, IQR −858 to +384 mL). Urinary sodium excretion significantly increased from 24 mmol/L (IQR: 10–53 mmol/L) to 85 mmol/L (IQR: 50–121 mmol/L) (paired t-test: mean difference: 42.9 mmol/L, 95% confidence interval [CI]: 22.0–63.7 mmol/L; *p* = 0.001) following the initiation of diuretics (Figure 1(A)). However, a significant reduction in 2-h CrCl was noted between baseline and day-2 after the initiation of diuretics (median: 94 mL/min vs. 42 mL/min; paired t-test: mean difference: −43.2 mL/min, 95%CI −12.7 to −73.8 mL/min; *p* = 0.007) (Figure 1(B)). The reduction in CrCl did not appear to be different between patients with and without sepsis. The significance of these results remained unchanged when using a non-parametric Wilcoxon Signed Rank test (*p* < 0.001). A reduction in 2-h CrCl occurred in 24 patients (77%) and was not related to the Sequential Organ Failure Assessment (SOFA) score prior to the initiation of diuretics (Mann-Whitney test: *p* = 0.502).

Using a linear mixed model that included total fluid balance (over the three study days), patient age, and baseline SOFA score, it was confirmed that SOFA was not associated with changes in CrCl (estimate: −0.303, 95%CI: −9.54 to 3.47; *p* = 0.347). However, both age (*p* = 0.048) and the quantity of negative fluid balance (*p* = 0.006) were significantly associated with changes in CrCl. None of the 31 study patients required dialysis or died during the hospital stay.

This observational study demonstrated that initiating loop diuretics, primarily furosemide, at relatively low doses was associated with significant natriuresis and negative fluid balance within 48 h in critically ill patients. Although plasma creatinine concentrations between the two time points were not significantly different (mean difference: 0.36 μmol/L, 95% CI: −15.1 to 15.8; *p* = 0.963), there was a significant reduction in 2-h CrCl during the same period. The lack of association between the SOFA score and the reduction in CrCl suggests that this reduction might be attributed to furosemide itself rather than the progression of critical illness. A previous study showed that administering furosemide (20 mg orally with 20 mg intravenously) could reduce glomerular filtration rate by 15 mL/min within three hours in healthy volunteers (*p* = 0.03) (and >25 mL/min among individuals with diastolic dysfunction) when measured using technetium-99m-diethylenetriaminepentaacetic acid (99mTc-DTPA) [8]. In our linear mixed model, the degree of negative balance was quantitatively associated with adverse changes in CrCl, suggesting that excessive negative balance over a short period could reduce CrCl in critically ill patients, although not to the extent that would affect survival or necessitate dialysis.

We must acknowledge the limitations of our study. It was observational with a small sample size, and we did not measure AKI biomarkers such as cystatin C and neutrophil gelatinase-associated lipocalin (NGAL) [4]. Circulating cystatin C is freely filtered at the glomerulus and reabsorbed by the proximal tubules, where it is completely catabolized. Thus, serum cystatin C concentration serves as a surrogate marker of glomerular filtration rate, while urinary cystatin C concentration may reflect impaired proximal tubular function [9]. Without AKI biomarker results, we could not be certain that the use of loop diuretics was causally related to the reduction in CrCl or whether furosemide could induce proximal tubular dysfunction. Despite a substantial reduction in CrCl within 48 h, none of the patients required dialysis, suggesting that the observed deteriorations in CrCl were transient and limited in severity. Additionally, our study only included critically ill patients, so the results may not be generalizable to less seriously ill patients.

In summary, this prospective cohort study showed that loop diuretics effectively induced negative fluid balance and natriuresis in critically ill patients. The diuresis and negative fluid balance induced by loop diuretics were temporarily associated with a transient reduction in CrCl in both septic and non-septic patients. A randomized controlled trial comparing loop diuretics with placebo, focusing on serial changes in CrCl and serum and urinary cystatin C concentrations over a longer duration, is needed to better understand the long-term renal impact of loop diuretics in critically ill patients, especially when high doses are used.
